# Three new species of the subgenus *Leipopleura* Seidlitz from Tibet, China (Coleoptera, Tenebrionidae, *Bioramix* Bates)

**DOI:** 10.3897/zookeys.609.8250

**Published:** 2016-08-08

**Authors:** Yun-Chun Li, L.V. Egorov, Ai-Min Shi

**Affiliations:** 1 College of Plant Protection, Southwest University, Beibei, Chongqing 400700, China; 2 The State Nature Reserve «Prisursky», Lesnoj, 9, Cheboksary 428034, Russia; 3 Key Laboratory of Southwest China Wildlife Resources Conservation, Institute of Rare Animals & Plants, China West Normal University, Nanchong, Sichuan 637009, China

**Keywords:** Darkling beetles, identification key, Platyscelidini, taxonomy

## Abstract

Three new species of darkling beetles (Tenebrionidae) belonging to the subgenus *Leipopleura* of the genus *Bioramix* Bates, 1879, Bioramix (Leipopleura) baqenensis Li & Egorov, **sp. n.**, Bioramix (Leipopleura) nyainrongensis Li & Egorov, **sp. n.**, and Bioramix (Leipopleura) banbarensis Li & Egorov, **sp. n.** are described from the Tibet Autonomous Region in China. Additionally, a new identification key is provided to all known Chinese representatives of the subgenus *Leipopleura*.

## Introduction

The genus *Bioramix* Bates, 1879 (Tenebrionidae: Platyscelidini) consists of approximately 115 species and it is subdivided into 13 subgenera. Only four subgenera, *Bioramix* Bates, 1879, *Cardiobioramix* Kaszab, 1940, *Leipopleura* Seidlitz, 1893 and *Tricholeipopleura* Kaszab, 1940 have been reported from China.

The subgenus *Leipopleura* with *Bioramix integra* Reitter, 1887 as the type species, is characterized by the intercoxal process not raised, sharply sloping behind procoxae; male abdomen medial setose patches absent; protibia underside concave at apex and their outer margin sharp, outer apical angles elongate; male pro- and mesotarsi widened, dense plantar pubescence present on proximal four tarsomeres. So far, 15 species have been described (Seidlitz 1893; Kaszab 1940; Egorov 1990, 2004, 2006a, b, 2008, 2009; Meng and Ren 2005; Li et al. 2013). Among these, 13 species were exclusively reported from China: *Bioramix aenescens* (Blair, 1923), *Bioramix politicollis* (Kaszab, 1940), *Bioramix kochi* (Kaszab, 1940), *Bioramix nagquana* (Meng & Ren, 2005) and *Bioramix igori* Li & L. Egorov, 2013 from Tibet; *Bioramix rubripes* (Reitter, 1889), *Bioramix rufipalpis* (Reitter, 1887) from Qinghai and Tibet; *Bioramix hongyuanensis* Li & L. Egorov, 2013 from Sichuan; *Bioramix frivaldszkyi* (Kaszab, 1940) from Gansu and Qinghai; *Bioramix integra* (Reitter, 1887) from Gansu, Qinghai and Sichuan; *Bioramix micans* (Reitter, 1889) from Gansu, Qinghai and Tibet; *Bioramix reinigi* (Kaszab, 1940) from Qinghai, Sichuan and Tibet. Meanwhile, *Bioramix crypticoides* (Reitter, 1887) from Gansu, Qinghai, Sichuan, Xingjiang and Tibet.

During the identification of tenebrionid specimens collected in Tibet in 2010, three new species of the subgenus *Leipopleura* were found and are described below.

## Material and methods

The specimens examined in this study are deposited in the Museum of China West Normal University, Nanchong, China (MCWNU) and Zoological Institute of Russian Academy of Sciences (ZIN) (St.-Petersburg, Russia). The specimens were examined with a Leica M205C stereomicroscope and recorded with a Nikon D3000 digital camera.

In the morphological descriptions the following measurements were taken (by means of binocular micrometre): 1) length of antennomeres (without interantennal membrane) and their maximum width; 2) length of pronotum along midline between anterior and posterior margins; 3) maximum width of pronotum; 4) length of elytra along suture from base to apex; 5) maximum width of elytra; 6) maximum width of tibia at apex; 7) maximum width of male tarsi; 8) maximum width and length of parameres (taken in dorsal view along middle groove); 9) length of phallobase (taken in lateral view) and general length of the aedeagus from apex of parameres to base of phallobase; 10) general length of the body from base of mandibles to apex of elytra (taken in lateral view). Density of punctation is characterized as follows: 1) dense punctation – distance between punctures less than their diameter; 2) moderately dense – distance between punctures less than or equal to their diameter; 3) sparse punctation – distance between punctures exceeding their diameter.

## Taxonomy

### Family Tenebrionidae Latreille, 1802 Subfamily Tenebrioninae Latreille, 1802 Tribe Platyscelidini Lacordaire, 1859 Genus *Bioramix* Bates, 1879

#### Bioramix (Leipopleura) baqenensis

Taxon classificationAnimaliaColeopteraTenebrionidae

Li & Egorov sp. n.

http://zoobank.org/8D6014AE-22A7-4F2D-BDA1-0A2975D15632

Figs 1–10 , 31–32

##### Type material.

Holotype: male, **CHINA**: Tibet, Baqen, 31°50.421'N, 094°18.016'E, 4575 m, 3 Jul. 2010, Ai-Min Shi and Yong-Sheng Pan leg. (MCWNU). Paratype: 21 males and 20 females, 4 paratypes (2 males, 2 females) in ZIN, same data as the holotype.

##### Diagnosis.

This new species can be distinguished based on the following: anterior margin of pronotum straight; protibia outer apical angles strongly elongated, inner surface with an obscure spur and strong setae at apical ½; parameres moderately narrowed apically, slightly sinuate near the apex ⅕ (in lateral view).

##### Etymology.

Named after the type locality, Baqen.

##### Description.

Body black; antennae, legs and palps brown; surface weakly shiny.

**Figures 1–10.**
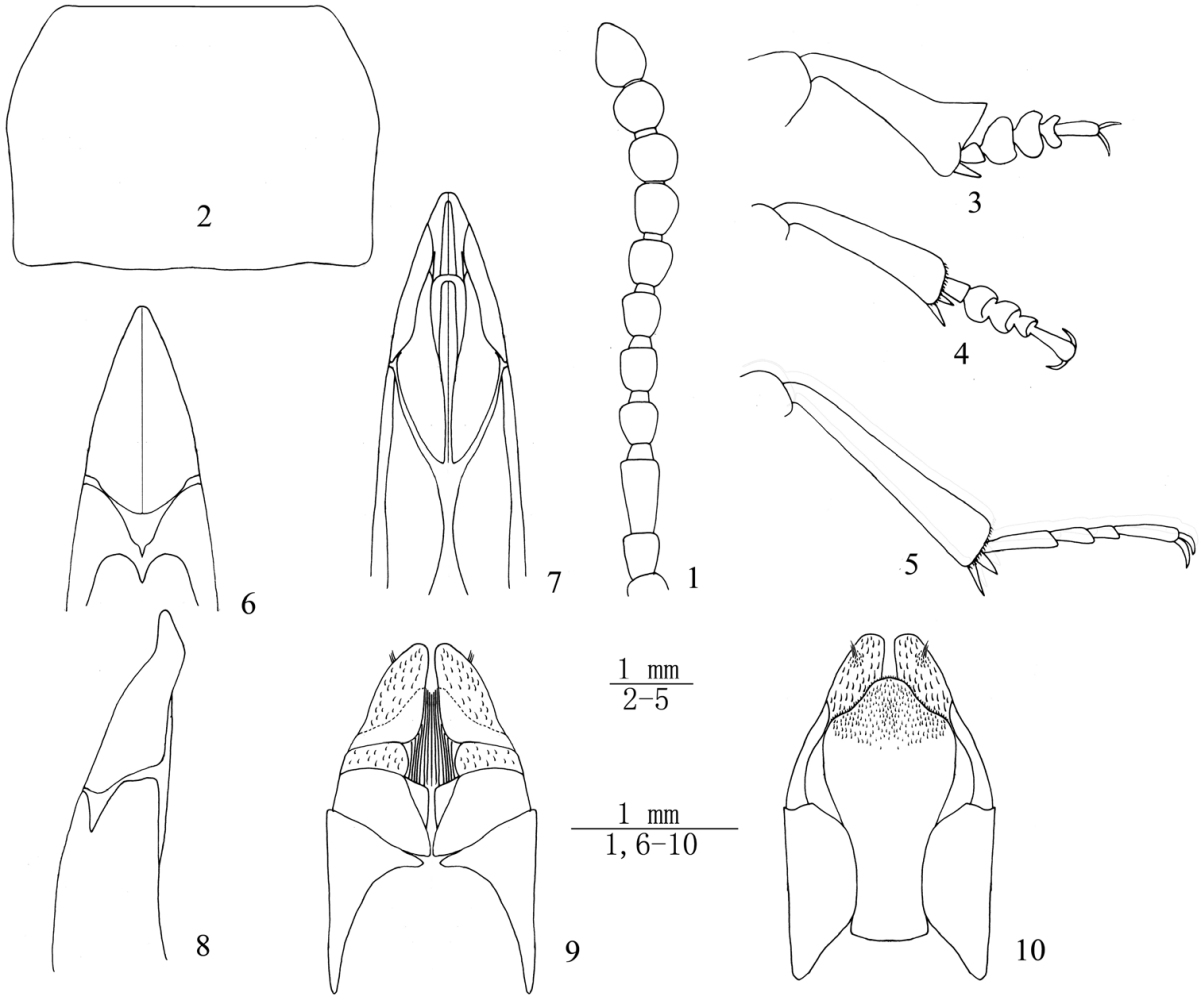
Bioramix (Leipopleura) baqenensis sp. n.: **1** antenna of male **2** pronotum of male **3** protibiae and protarsomeres of male **4** mesotibiae and mesotarsomeres of male **5** metatibiae and metatarsomeres of male **6–7** apical part of aedeagus in dorsal and ventral views **8** apical part of aedeagus in lateral view **9–10** ovipositor in dorsal and ventral views.

**Male** (Figs 1–8). *Head* broad, anterior margin of clypeus straight, fronto-clypeal suture slightly obscure; most of genae densely punctate, covered with recumbent hairs. Dorsal surface of head slight convex. Punctation of head fine, dense or moderately dense. Eyes transverse, with shallow emargination at anterior margin. Antennae when posteriorly extended, reaching pronotal base. Length (width) ratio of antennomeres from 2^nd^ to 11^th^ as follows: 14.0(11): 25.5(12.5): 14.0(12.5): 13.5(11.5): 15.0(12.0): 14.5(13.0): 17.5(15.0): 15.5(15.0): 15.5(15.5): 20.5(16.0) (n = 5).

*Pronotum* (Fig. 2) transverse, 1.35–1.51 (1.44 on average, n = 5) times as wide as long, widest before the middle, 1.72–1.82 (1.78 on average, n = 5) times as wide as head. Ratio of pronotal width at anterior margin to its maximum width and width at base (n = 5) 0.69: 1.00: 0.95 on average. Outer margin of pronotum shallowly sinuate in basal ½, more abruptly converging anteriorly in anterior ⅓, finely bordered along entire length. Anterior margin straight, bordered laterally; base weakly bisinuate, not bordered or bordered laterally. Anterior angles of pronotum widely obtuse; posterior ones nearly rectangular. Pronotal surface between outer margins convex, punctures larger and denser than those on head, finer at disc than laterally, lateral margins of pronotum weakly flattened. Intercoxal process not raised, sharply sloping behind procoxae. Prothoracic hypomeron with longitudinal wrinkles.

*Elytra* elongate-oval, 1.32–1.43 (1.36 on average, n=5) times as long as wide, maximum width before middle, 1.23–1.27 (1.25 on average, n=5) times as wide as pronotum. Outer margin of epipleural reaching sutural angle, visible dorsally only at base. Elytral surface between epipleura and sutural margin convex, with fine dense punctation and minute rugae, with traces of longitudinal prominences and also traces of smooth rounded prominences better developed in apical half. Epipleural surface with densely covered irregular wrinkles and small granules. Mesoventrite with rather sparse recumbent hairs; surface finely granulate and wrinkles. Abdominal ventrites with yellow hairs. Intercoxal process of abdomen rounded apically. First and second abdominal ventrites with shallow medial impressions, 4^th^ ventrite weakly concave at sides. Last abdominal ventrite with shallow depression and impunctate semicircular area at medial base.

*Legs* (Figs 3–5) robust, length (width) ratio of pro-, meso- and metafemora 69.0(25.0): 73.5(22.5): 88.0(24.5) (n = 5); that of corresponding tibiae 67.0(27.0): 63.5(17.5): 88.0(18.0) (n = 5). Protibiae 2.37–2.59 (2.49 on average, n = 5) times as long as wide, gradually widening in basal ⅔. Outer apical angles strongly elongate, underside concave. Inner surface with an obscure spur and strong setae in apical ½. Metatibia straight. Plantar surface of proximal pro- and mesotarsomeres 1-4 with setal brushes. Length (width) ratio of pro-, meso- and metatarsomeres from 1^st^ to 4^th^ as follows: 8.0 (7.0): 11.0 (15.5): 8.5 (14.5): 4.5 (12.5) (n = 5), 10.0 (8.0): 9.5(12.5): 7.0 (11.0): 5.0 (7.5) (n = 5) and 27.5 (6.5): 13.5 (6.0): 11.5 (5.5): 21.0 (5.0) (n = 5).

*Aedeagus* (Figs 6–8): length 2.9–3.0 mm, width 0.83 mm. Parameres 1.17 mm long and 0.67 mm wide, moderately narrowed apically, apical ⅕ slightly sinuate (in lateral view).

**Female** (Figs 9–10). Body wider. Antennae and epipleural carina shorter than in male. Pronotum 1.60–1.72 (1.65 on average, n = 5) times as wide as long, 1.76–1.81 (1.78 on average, n = 5) times as wide as head. Ratio of pronotal width at anterior margin to its maximum width and width at base 0.71: 1.00: 0.95. Elytra 1.27–1.35 (1.32 on average, n = 5) times as long as wide, 1.29–1.40 (1.35 on average, n = 5) times as wide as pronotum. Plantar surface of pro- and mesotarsomeres without setal brush. Ovipositor in dorsal view with golden setae at apical ⅓, tuft of setae (with 3–4 long setae) present near apical margin.

##### Measurements.

Male body length 9.3–10.4 mm, width 4.7–5.0 mm; female body length 9.1–9.8 mm, width 4.7–5.2 mm.

##### Distribution.

China: Tibet (Tanggula Shan, Baqen).

##### Remarks.

Bioramix (Leipopleura) baqenensis Li & Egorov, sp. n. is similar to Bioramix (Leipopleura) nagquana (Meng & Ren, 2005) based on the following characters: length of antennae (when posteriorly extended, reaching pronotal base), anterior margin of clypeus straight, pronotum widest near middle, and metatibia straight.

#### Bioramix (Leipopleura) nyainrongensis

Taxon classificationAnimaliaColeopteraTenebrionidae

Li & Egorov sp. n.

http://zoobank.org/36338F33-8DD8-48DF-B594-522B93D95AC1

Figs 11–20 , 33–34

##### Type material.

Holotype: male, **CHINA**: Tibet, Nyainrong, 32°06.763'N, 92°17.171'E, 4728 m, 6 Aug. 2010, Yun-Chun Li and Yong-Sheng Pan leg. (MCWNU). Paratype: 4 males and 3 females, 3 paratypes (2 males, 1 females) in ZIN, same data as the holotype.

##### Diagnosis.

This new species can be distinguished based on the following: shorter antennae (when posteriorly extended, not reaching pronotal base); anterior margin of pronotum emarginated, and metatibia weakly incurved.

##### Etymology.

Named after the type locality, Nyainrong.

##### Description.

Body black; antennae, legs and palps brown; surface weakly shiny.

**Figures 11–20.**
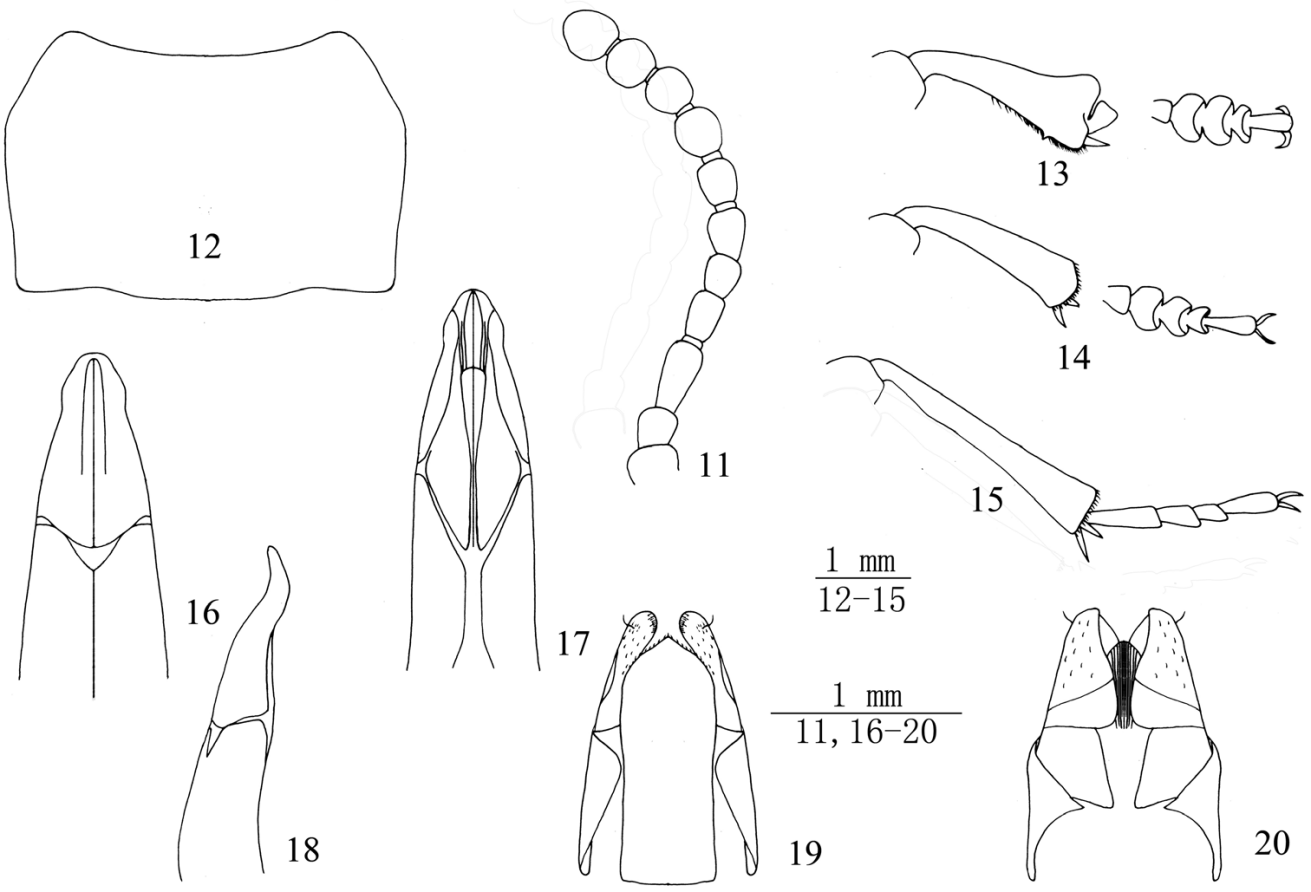
Bioramix (Leipopleura) nyainrongensis sp. n.: **11** antenna of male **12** pronotum of male **13** protibiae and protarsomeres of male **14** mesotibiae and mesotarsomeres of male **15** metatibiae and metatarsomeres of male **16–17** apical part of aedeagus in dorsal and ventral views **18** apical part of aedeagus in lateral view **19–20** ovipositor in dorsal and ventral views.

**Male** (Figs 11–18). *Head* broad, anterior margin of clypeus weakly, but noticeably arcuate in the middle; fronto-clypeal suture slightly obscure; most of genae densely punctate, covered with recumbent hairs. Dorsal surface of head slight convex. Punctation of head coarse large, dense or moderately dense. Antennae very shorter, when posteriorly extended, not reaching posterior ½ of pronotum. Length (width) ratio of antennomeres from 2^nd^ to 11^th^ as follows: 11.7(10.3): 20.7(10.0): 12.7(9.3): 12.3(10.0): 13.7(10.3): 13.3(10.3): 14.0(13.3): 12.0(13.0): 13.0(14.0): 17.0(14.7) (n = 3).

*Pronotum* (Fig. 12) transverse, 1.49–1.63 (1.55 on average, n = 3) times as wide as long, widest before in the middle, 1.75–1.87 (1.83 on average, n = 3) times as wide as head. Ratio of pronotal width at anterior margin to its maximum width and width at base (n = 3) 0.66: 1.00: 0.96 on average. Outer margins of pronotum acutely convex, bordered along entire length. Anterior margin emarginate, bordered laterally; base weakly bisinuate, not bordered or bordered laterally. Anterior angles weakly obtuse; posterior ones nearly rectangular. Pronotal surface between outer margins convex, punctures smaller and denser than those on head, finer at disc than laterally, lateral margins of pronotum weakly flattened. Intercoxal process not raised, sharply sloping behind procoxae. Prothoracic hypomeron with longitudinal wrinkles.

*Elytra* elongate-oval, 1.27–1.33 (1.30 on average, n = 3) times as long as wide, maximum width before middle, 1.10–1.20 (1.14 on average, n = 3) times as wide as pronotum. Outer margin of epipleural reaching sutural angle, visible dorsally only at base. Elytral surface between epipleura and sutural margin convex, with traces of longitudinal carina, elytra apex sharply declined. Epipleural surface covered with dense irregular wrinkles and sparse shallow punctures. Lateral carina of elytra (outer margin of pseudepipleura) visible in dorsal view only anteriorly, explanate on humeri, merging with epipleura, reaching sutural angle. Mesoventrite with rather sparse recumbent hairs; surface finely granulate and wrinkles. Abdominal ventrites with yellow hairs. intercoxal process of abdomen rounded apically. First and second abdominal ventrites with shallow medial impressions.

*Legs* (Figs 13–15), length (width) ratio of pro-, meso- and metafemora 53.3(19.7): 59.7(18.7): 70.7(19.3) (n = 3); that of corresponding tibiae 53.3(20.7): 54.3(14.0): 72.7(14.3) (n = 3). Protibiae 2.55–2.60 (2.58 on average, n = 3) times as long as wide, gradually widening towards apex, Outer apical angles weakly elongate, underside concave. pro-, meso- and metatibiae with densely golden hairs at apex of inner surface, protibiae with obviously a spur. metatibia weakly incurved. Plantar surface of proximal pro- and mesotarsomeres 1-4 with setal brushes. Length (width) ratio of pro-, meso- and metatarsomeres from 1^st^ to 4^th^ as follows: 8.0(6.7): 7.7 (13.3): 6.3 (13.7): 4.7 (10.0) (n = 3), 8.0 (7.0): 8.0 (12.3): 6.3 (10.0): 4.7 (6.3) (n = 3) and 21.7 (6.7): 10.3 (6.0): 8.3 (5.7): 16.0 (6.0) (n = 3).

*Aedeagus* (Figs 16–18): length 2.4–2.5 mm, width 0.67 mm. Parameres 0.95 mm long and 0.57 mm wide. Parameres noticeably narrowed toward apex in dorsal view, but weakly widened in distal ¼, distinctly sinuate (in lateral view).

**Female** (Figs 19–20). Body longer and wider. Pronotum 1.57–1.59 (1.58 on average, n = 3) times as wide as long, 1.75–1.77 (1.76 on average, n = 3) times as wide as head. Ratio of pronotal width at anterior margin to its maximum width and width at base (n = 3) 0.67: 1.00: 0.98. Elytra 1.30–1.32 (1.31 on average, n = 3) times as long as wide, 1.19–1.21 (1.20 on average, n = 3) times as wide as pronotum. Plantar surface of pro- and mesotarsomeres without setal brush. Ovipositor in dorsal view with golden setae at apical ¼, and nearly apex shorter setae formation looped pile, within a strong long setae highlight, inner apical surface of with densely setae.

##### Measurements.

Male body length 7.9–8.1 mm, width 3.9–4.0 mm; female body length 8.1–8.2 mm, width 4.0–4.1 mm.

##### Distribution.

China: Tibet (Tanggula Shan, Nyainrong).

##### Remarks.

Bioramix (Leipopleura) nyainrongensis Li & Egorov, sp. n. is similar to Bioramix (Leipopleura) baqenensis Li & Egorov, sp. n. and Bioramix (Leipopleura) nagquana (Meng & Ren, 2005) based on the following characters: pronotum (widest near middle), posterior margin of pronotum base weakly bisinuate, elytra elongate-oval (widest near middle), and outer margin of epipleural visible dorsally only at base.

#### Bioramix (Leipopleura) banbarensis

Taxon classificationAnimaliaColeopteraTenebrionidae

Li & Egorov sp. n.

http://zoobank.org/4CB6BCA8-16DA-4125-99BA-953536EC0519

Figs 21–30 , 35–36

##### Type material.

Holotype: male, **CHINA**: Tibet, Banbar, 30°55.964'N, 094°42.482'E, 3730 m, 25 Jul. 2010, Ai-Min Shi and Yong-Sheng Pan leg. (MCWNU). Paratype: 59 males and 51 females, 4 paratypes (2 males, 2 females) in ZIN, same data as the holotype.

##### Diagnosis.

This new species can be distinguished based on the following: shorter antennae (when posteriorly extended, not reaching pronotal base), anterior margin of pronotum straight, parameres distinctly sinuate near the apex ⅙ (in lateral view).

##### Etymology.

Named after the type locality, Banbar.

##### Description.

Body dark brown; surface weakly shiny.

**Figures 21–30.**
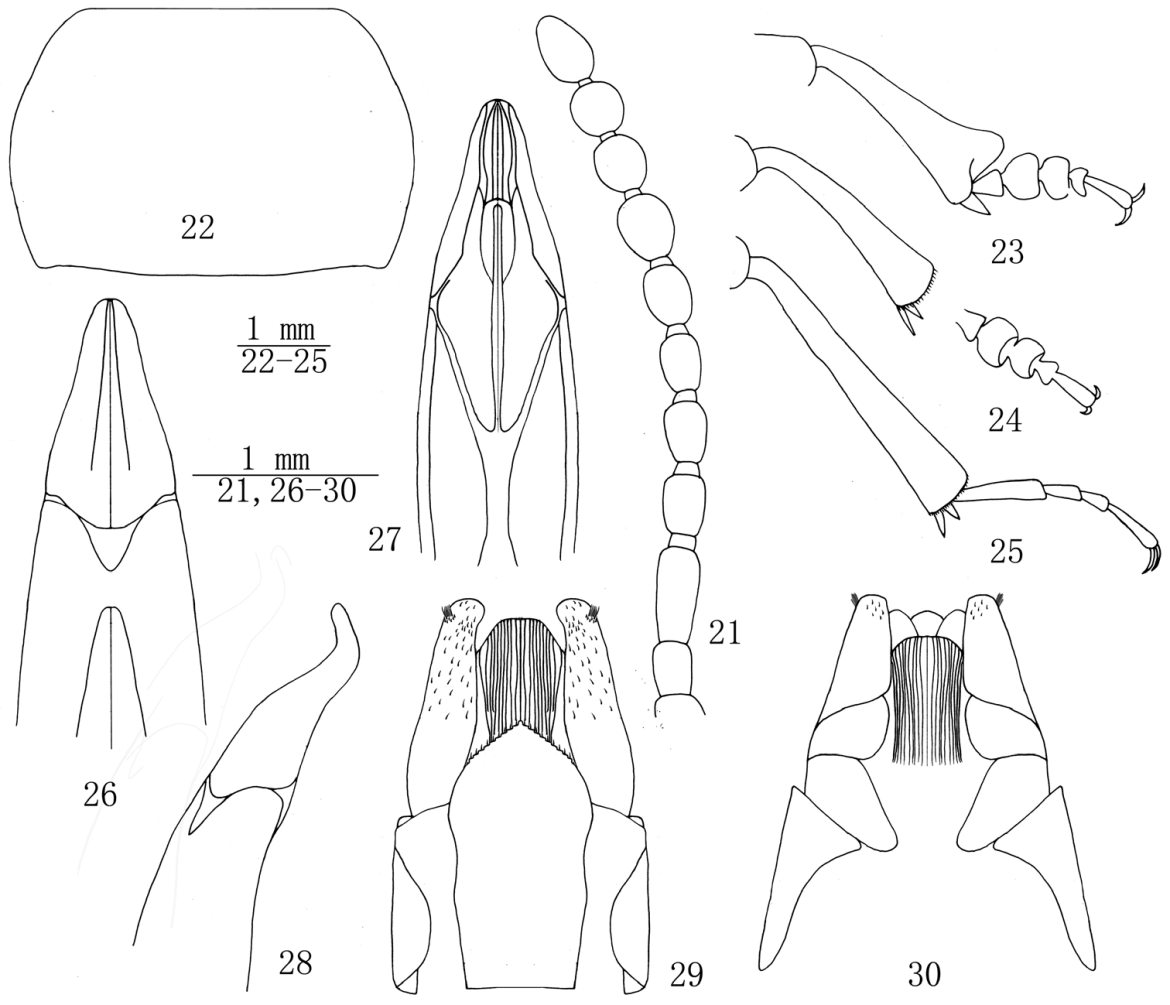
Bioramix (Leipopleura) banbarensis sp. n.: **21** antenna of male **22** pronotum of male **23** protibiae and protarsomeres of male **24** mesotibiae and mesotarsomeres of male **25** metatibiae and metatarsomeres of male **26–27** apical part of aedeagus in dorsal and ventral views **28** apical part of aedeagus in lateral view **29–30** ovipositor in dorsal and ventral views.

**Male** (Figs 21–28). *Head* broad, anterior margin of clypeus straight, fronto-clypeal suture slightly obscure; most of genae densely punctate, covered with recumbent hairs. Dorsal surface of head slight convex. Punctation of head fine, dense or moderately dense. Eyes transverse, with shallow emargination at anterior margin. Antennae short, when posteriorly extended, not apices reaching base of pronotum. Length (width) ratio of antennomeres from 2^nd^ to 11^th^ as follows: 15.8(11.6): 27.2(12.2): 18.4(12.0): 17.4(12.0): 17.6(12.0): 16.8(12.2): 18.8(15.6): 15.8(14.8): 15.8(14.6): 22.4(14.0) (n = 5).

**Figures 31–36.**
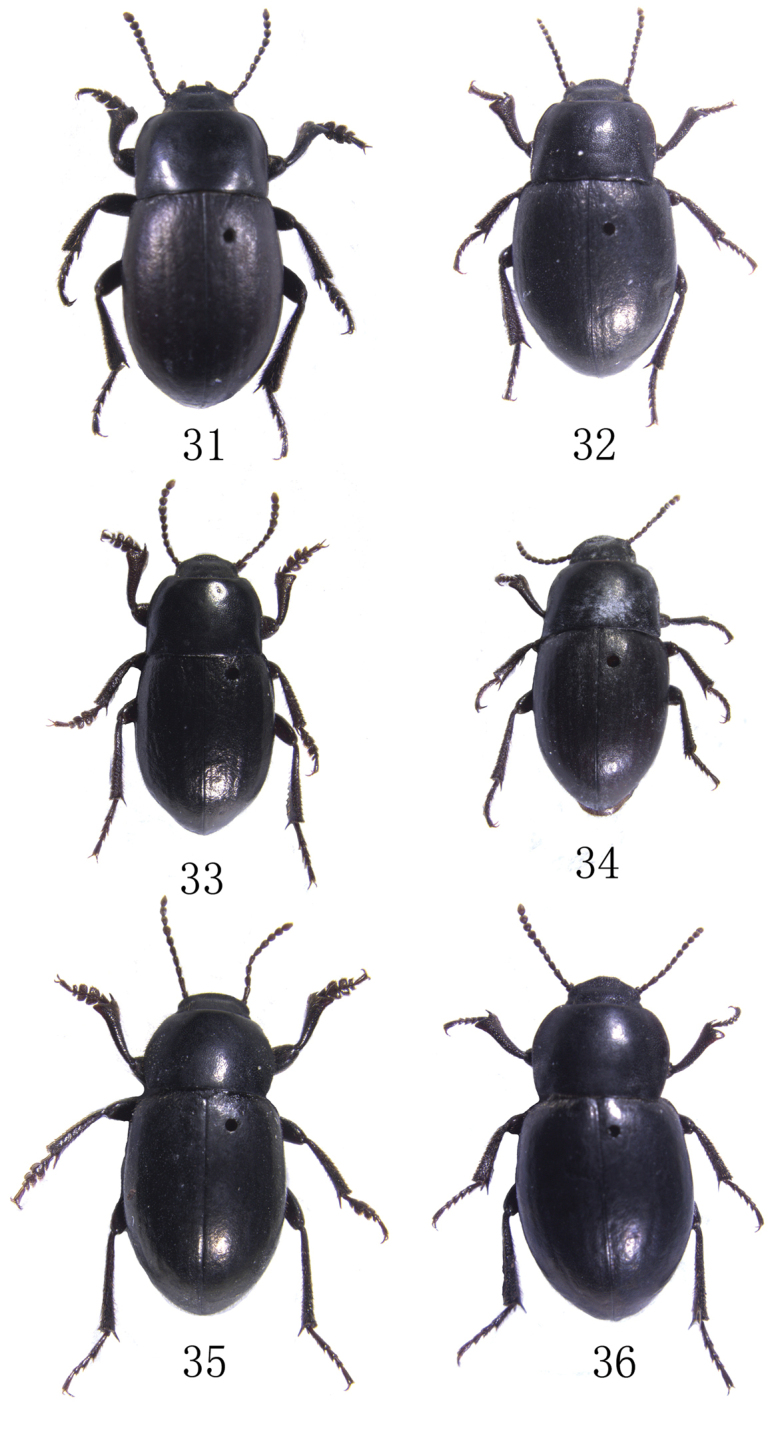
**31–32** Bioramix (Leipopleura) baqenensis sp. n.: **31** male, length 9.3–10.4 mm **32** female, length 9.1–9.8 mm **33–34** Bioramix (Leipopleura) nyainrongensis sp. n.: **33** male, length 7.9–8.1 mm **34** female, length 8.1–8.2 mm **35–36** Bioramix (Leipopleura) banbarensis sp. n.: **35** male, length 9.9–10.6 mm **36** female, length 10.0–10.5 mm.

*Pronotum* (Fig. 22) transverse, 1.41–1.51 (1.47 on average, n = 5) times as wide as long, widest in the middle, 1.69–1.79 (1.75 on average, n = 5) times as wide as head. Ratio of pronotal width at anterior margin to its maximum width and width at base (n = 5) 0.64: 1.00: 0.88 on average. Outer margins of pronotum acutely convex, bordered along entire length. Anterior margin straight, bordered laterally; base weakly bisinuate, not bordered. Anterior angles of pronotum widely obtuse, posterior ones weakly obtuse. Pronotal surface between outer margins convex, with trace of slight depression. Punctuation on disc similar to that on frons, mainly dense, coarser and denser at sides. Prosternum with rather sparse hairs directed backwards and small granules. Intercoxal process not raised, sharply sloping behind procoxae. Prothoracic hypomeron with obvious longitudinal wrinkles and coarse granules.

*Elytra* elongate-oval, 1.28–1.45 (1.35 on average, n = 5) times as long as wide, widest nearly in the middle, 1.21–1.34 (1.28 on average, n = 5) times as wide as pronotum. Outer margin of epipleural not reaching sutural angle, visible dorsally only at base. Elytral surface between epipleura and sutural margin convex, with sparse and shallow punctures, irregular fine wrinkles and traces of longitudinal carina. Elytra margin setae and abdomen setae subequal length, elytra apex sharply declined. Mesosternum with rather sparse recumbent hairs, surface finely granulate and wrinkles. Abdominal ventrites with yellow hairs. First and second abdominal ventrites with shallow medial impressions. Last abdominal ventrite with depression and impunctate semicircular area at medial base, apical margin widely rounded.

*Legs* (Figs 23–25) robust, length (width) ratio of pro-, meso- and metafemora 70.4(25.0): 76.2(23.0): 93.2(24.6) (n = 5); that of corresponding tibiae 68.4(25.6): 68.6(19.4): 98.4(19.2) (n = 5). Protibiae 2.45–2.95 (2.78 on average, n = 5) times as long as wide, gradually widening towards apex, apical margin extended but not forming sharp triangle, underside concave. Pro- and mesotibia subequal length. Protibia with an obscure spur, metatibia nearly straight. Plantar surface of proximal pro- and mesotarsomeres 1-4 with setal brushes. Length (Width) ratio of pro-, meso- and metatarsomeres from 1^st^ to 4^th^ as follows: 8.6(8.2): 10.4 (17.4): 8.2 (16.0): 5.4 (9.8) (n = 5), 9.2 (8.2): 9.6 (15.4): 8.4 (12.8): 6.0 (7.4) (n = 5) and 28.2 (7.8): 13.4 (6.6): 10.6 (6.0): 21.6 (5.8) (n = 5).

*Aedeagus* (Figs 26–28): length 3.1–3.2 mm, width 0.93 mm. Parameres 1.15 mm long and 0.70 mm wide, moderately narrowed apically, apical ⅙ distinctly sinuate (in lateral view).

**Female** (Figs 29–30). Body longer and wider. Pronotum 1.50–1.57 (1.54 on average, n = 5) times as wide as long, 1.79–1.88 (1.82 on average, n = 5) times as wide as head. Ratio of pronotal width at anterior margin to its maximum width and width at base (n = 5) 0.70: 1.00: 0.95. Elytra 1.32–1.39 (1.35 on average, n = 5) times as long as wide, 1.24–1.34 (1.28 on average, n = 5) times as wide as pronotum. Plantar surface of pro- and mesotarsomeres without setal brush. Ovipositor in dorsal view with golden setae at apical ⅖, tuft of setae (with 7–8 long setae) present near apical margin.

##### Measurements.

Male body length 9.9–10.6 mm, width 4.9–5.4 mm; female body length 10.0–10.5 mm, width 5.0–5.4 mm.

##### Distribution.

China: Tibet (Nyainqentanglha Shan (Nyenchen Tanglha Mountains), Banbar).

##### Remarks.

Bioramix (Leipopleura) banbarensis Li & Egorov, sp. n. is similar to Bioramix (Leipopleura) crypticoides (Reitter, 1887) based on the following characters: pronotum widest near middle, anterior and posterior angles of pronotum obtuse, base weakly bisinuate, protibia apical margin extended not sharp apices, and metatibia nearly straight.

##### Conclusion.

The world fauna of the tribe Platyscelidini comprises 8 genera, 28 subgenera (Egorov 2004, 2009) and found in Palaearctic area. moreover, its southern border coincides with the part of Palaearctic southern border in the Himalayas. The majority of species live in the steppe and mountain habitats, lesser number in semideserts. The mountain regions of Asia, such as the Tien Shan, the Pamirs, the Hindu Kush, the Karakorum Range, the unlun Shan, the Himalayas, as well as Northern and Central China mountains are the main centers of species diversity. The classification of the tribe, developed in detail by Kaszab (1940) and Egorov (2004, 2009).

### Key to the species of the subgenus *Leipopleura* from China

**Table:** 

1	Pronotum widest near or at base	**2**
–	Pronotum widest anterior to or at middle	**5**
2	Only basal part of the outer margin of epipleura visible dorsally	**3**
–	Anterior ⅓ or half of the outer margin of epipleura visible dorsally	**4**
3	Elytra widest at base. Metatibia slightly incurved	***Bioramix igori* Li & L. Egorov, 2013**
–	Elytra widest at middle. Metatibia straight	***Bioramix aenescens* (Blair, 1923)**
4	Anterior and basal margin of pronotum straight, anterior angles obtuse, posterior angles rectangular	***Bioramix politicollis* (Kaszab, 1940)**
–	Anterior margin of pronotum weakly sinuate, Pronotal base straight, anterior and posterior angles nearly rectangular	***Bioramix frivaldszkyi* (Kaszab, 1940)**
5	Outer margin of epipleura not reaching sutural angle	**6**
–	Outer margin of epipleura reaching sutural angle	**9**
6	Anterior margin of pronotum sinuate, base weakly bisinuate	***Bioramix nyainrongensis* sp. n.**
–	Anterior margin and base of pronotum straight	**7**
7	Parameres noticeably narrowed toward apex in dorsal view, but strongly widened in distal ⅕, with very flat apices	***Bioramix rufipalpis* (Reitter, 1887)**
–	Parameres subparallel, gradually narrowing towards apex, not widened distally, with moderately sharp apices	**8**
8	Parameres (in lateral view) strongly curved in distal ⅓	***Bioramix reinigi* (Kaszab, 1940)**
–	Parameres (in lateral view) weakly curved in distal ¼, nearly straight	***Bioramix crypticoides* (Reitter, 1887)**
9	Anterior margin of pronotum weakly sinuate	**10**
–	Anterior margin of pronotum straight	**12**
10	Pronotal base emarginate, elytra widest at the base	***Bioramix hongyuanensis* Li & L. Egorov, 2013**
–	Pronotal base nearly straight, elytra widest at the middle	**11**
11	Anterior and posterior angles of pronotum obtuse, legs red	***Bioramix rubripes* (Reitter, 1889)**
–	Anterior angles of pronotum obtuse, posterior angles sharp and rectangular, legs brown	***Bioramix nagquana* (Meng & Ren, 2005)**
12	Anterior margin of clypeus straight	**13**
–	Anterior margin of clypeus weakly, but noticeably, arcuate in the middle	**15**
13	Anterior angles of pronotum very rounded, elytra base and pronotum almost as wide	***Bioramix kochi* (Kaszab, 1940)**
–	Anterior angles of pronotum not rounded, elytra base wider than pronotum	**14**
14	Antenna short, when posteriorly extended, not reaching pronotal base. Outer apical angles of protibiae weakly elongated	***Bioramix banbarensis* sp. n.**
–	Antenna long, when posteriorly extended, reaching pronotal base. Outer apical angles of protibiae strongly elongated	***Bioramix baqenensis* sp. n.**
15	Pronotal punctation coarse, parameres almost straight in lateral view	***Bioramix micans* (Reitter, 1889)**
–	Pronotal punctation fine, parameres noticeably curved in distal ¼ in lateral view	***Bioramix integra* (Reitter, 1887)**

## Supplementary Material

XML Treatment for Bioramix (Leipopleura) baqenensis

XML Treatment for Bioramix (Leipopleura) nyainrongensis

XML Treatment for Bioramix (Leipopleura) banbarensis
